# The Impact of Partial Measurement Invariance on Testing Moderation for Single and Multi-Level Data

**DOI:** 10.3389/fpsyg.2018.00740

**Published:** 2018-05-15

**Authors:** Yu-Yu Hsiao, Mark H. C. Lai

**Affiliations:** ^1^Center on Alcoholism, Substance Abuse, and Addictions, University of New Mexico, Albuquerque, NM, United States; ^2^School of Education, University of Cincinnati, Cincinnati, OH, United States

**Keywords:** measurement equivalence, measurement invariance, moderation, interaction effects, structural equation modeling, hierarchical linear modeling, multilevel modeling

## Abstract

Moderation effect is a commonly used concept in the field of social and behavioral science. Several studies regarding the implication of moderation effects have been done; however, little is known about how partial measurement invariance influences the properties of tests for moderation effects when categorical moderators were used. Additionally, whether the impact is the same across single and multilevel data is still unknown. Hence, the purpose of the present study is twofold: (a) To investigate the performance of the moderation test in single-level studies when measurement invariance does not hold; (b) To examine whether unique features of multilevel data, such as intraclass correlation (ICC) and number of clusters, influence the effect of measurement non-invariance on the performance of tests for moderation. Simulation results indicated that falsely assuming measurement invariance lead to biased estimates, inflated Type I error rates, and more gain or more loss in power (depends on simulation conditions) for the test of moderation effects. Such patterns were more salient as sample size and the number of non-invariant items increase for both single- and multi-level data. With multilevel data, the cluster size seemed to have a larger impact than the number of clusters when falsely assuming measurement invariance in the moderation estimation. ICC was trivially related to the moderation estimates. Overall, when testing moderation effects with categorical moderators, employing a model that accounts for the measurement (non)invariance structure of the predictor and/or the outcome is recommended.

Many theories in education and psychology rely on moderators, which in Baron and Kenny's (1986) words, “[affect] the direction and/or strength of the relation between an independent or predictor variable and a dependent or outcome variable” (p. 1,174). For many years, social and behavioral researchers are interested in understanding whether a specific moderation effect occurs as well as what factors may influence the extent of the moderation effect. Numerous methodological studies regarding different aspects of moderation effects have been done in contexts such as multiple regression (Aiken and West, 1991), multiple-group structural equation modeling (multiple-group SEM; Jaccard and Wan, 1996), latent variable models with observed composites (Bohrnstedt and Marwell, 1978; Busemeyer and Jones, 1983; Hsiao et al., 2018), within-subject designs (Judd et al., 1996, 2001), cross-level interactions (Kreft et al., 1995), and Bayesian estimations (Lüdtke et al., 2013).

Much of the methodological research regarding moderation effects focused on continuous variables, and less research has been done for categorical moderators. As an example of the latter, researchers may be interested in how the effect of social support on happiness differs by gender. Gender as a categorical variable is treated as the moderator, and social support and happiness are the predictor and outcome variables, respectively. In testing such a moderation with conventional methods such as multiple regression and multiple-group SEM, researchers implicitly assume that the predictor and the outcome variables are measurement invariant across the categorical moderators; that is, the measurement characteristics for social support and happiness are the same by different gender categories. However, such an assumption is seldom investigated before testing moderation effects. Additionally, little is known about how measurement non-invariance influences the estimation of the moderation effects. Hence, it is worth investigating whether measurement invariance for both the predictor and the outcome variables with respect to the moderator categories is a necessary prerequisite before conducting a moderation effect testing.

Measurement invariance (MI) is an important issue in a variety of social and behavioral research settings, especially when the data are collected from multiple populations (Millsap and Kwok, 2004). Full MI holds when individuals with identical ability but from different groups have the same propensity to get a particular score on that specific ability scale (Yoon and Millsap, 2007). Under the multiple-group confirmatory factor analysis framework, a simplified but commonly used version of MI analyses can be conducted by testing four models with hierarchical orders across groups: equal model structures (configural invariance), equal factor loadings (metric invariance), equal intercepts (scalar invariance), and equal unique factor variances (strict invariance; Vandenberg and Lance, 2000; Millsap and Kwok, 2004; Chen et al., 2005; Brown, 2015). Among the four types of MI, metric invariance has been suggested as one basic requirement for doing prediction (Vandenberg and Lance, 2000), which is closely related to moderation effect as moderation effect is about the difference in path coefficients across groups. Hence, in this paper we focus on the impact of metric non-invariance on the estimation of moderation effects. We also focus on testing moderation effects with the multiple-group approach, which is generally being used for examining measurement invariance.

## Previous research on the effect of metric non-invariance on prediction

Millsap (1995, 1997, 1998, 2007) delineated several theorems and corollaries for the relationship between MI and prediction bias. Donahue (2006) conducted a simulation study to examine the change of the prediction accuracy when the measure of the exogenous (predictor) variable was non-invariant in some part of the factor loadings, or with the presence of partial metric invariance, across groups. Her study found that, if one correctly assumes a partial invariance model on the latent predictors' structures, the path coefficient estimates on the outcome variables are unbiased even with a larger degree of metric non-invariance (i.e., more non-invariant items) on the latent predictors. However, the study only included the effects on tests of simple regression coefficient in each group, but not moderation, which can be defined as the difference in path coefficients across groups. Additionally, the study did not show the consequences of failing to correctly model the non-invariance structure.

Guenole and Brown (2014) used Monte Carlo studies to investigate the impact of ignoring measurement invariance (including metric invariance) on testing linear and nonlinear effects (including moderation effects). They adopted relative bias of the estimated path coefficients and 95% coverage rate of the estimated confidence intervals from both the reference group and focal group. They found biased estimates of the path coefficients from the two groups when two or more (out of six) ignored non-invariant loadings occurred. The same results were observed when the non-invariance occurred for predictors and outcomes^1^.

In the present research, we address two gaps from the work of Donahue (2006) and Guenole and Brown (2014). First, we would show the degree to which estimations and tests of moderation are affected when researchers incorrectly assume that (metric) MI holds. Second, we are interested in whether the location of measurement non-invariance, particularly in the predictor or in the outcome variable, makes a difference. Furthermore, we extend their work by investigating the Type I error rate of misidentifying null moderation effect and the statistical power of detecting nonzero moderation effects in the presence of non-invariance.

Additionally, Donahue (2006) and Guenole and Brown (2014) focused on single level data structure, in which all the observations were assumed to be independent. However, educational and psychological data often have nesting structures (e.g., students nested within classrooms; Kim et al., 2012). For example, a researcher is interested in how the association between students' motivation and their academic achievement differs in public and private schools. Since students are nested within schools, the school variable is a moderator defined in the between level and motivation is a predictor defined in the within level. Therefore, the scenario represents a “cross-level” moderation effects. In this situation, the measurement characteristics of motivation and academic achievement are assumed invariant across school types (i.e., public vs. private). It is still unclear that how multilevel measurement metric (non)invariance across groups in the between level influences the cross-level moderation effects. Therefore, we also show how unique features of multilevel data affect the MI-moderation relationship^2^.

## Study 1

**Figure 1:**
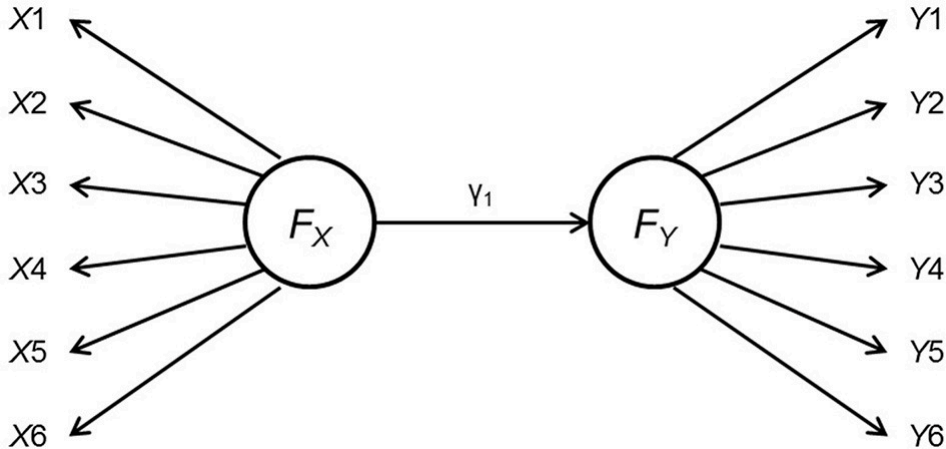
Data generating model for Study 1. *F*_*X*_ and *F*_*Y*_ are the latent predictor and outcome variables, each indicated by six observed indicators.

In Study 1, we aim to show the effect of measurement non-invariance on the power and Type I error rate when testing a moderator with two categories. Both the predictor and the outcome have a measurement structure and the moderation effects are tested with multiple-group approach, as shown in Figure 1. Specifically,

$$\begin{array}{l} {\text{X}}_{g}=\lambda_{\text{X}g}F_{\text{X}g}+\delta_{g},\\ {\text{Y}}_{g}=\lambda_{\text{Y}g}F_{\text{Y}g}+\varepsilon_{g},\\ F_{\text{Y}g}=\gamma_{g}F_{\text{X}g}+\zeta_{g}, \end{array}$$

where *g* = 1, 2 was the group index number, $\text{X}={\left[X_{1},X_{2},\ldots \right]}^{\prime }$ and $\text{Y}={\left[Y_{1},Y_{2},\ldots \right]}^{\prime }$ were observed indicators as shown in Figure 1, **λ**_X_ and **λ**_Y_ were two vectors of factor loadings of the indicators on the latent variables, **δ** and **ε** were vectors of the effects of unique factor on **X** and **Y**, γ_*g*_ is the path coefficient between *F*_X_ and *F*_Y_ for group *g*, and ζ was the latent disturbance term for *F*_Y_. In addition, both the impacts of having metric non-invariance on the outcome and on the predictor were investigated. The simulation study was described below.

### Monte carlo simulation

The study had a 3 (*p*_ni_, number of non-metric-invariant indicators) × 4 ($\gamma ={\left\{\gamma_{1},\gamma_{2}\right\}}^{\prime }$, vector of population regression coefficients of the two groups) × 2 (location of non-invariance) × 2 (*N*, sample size of each group) design. In each condition there were two groups, and the sample sizes were assumed equal across groups. Both the predictor *F*_X_ and the outcome *F*_Y_ were latent variables with six indicators.

#### Number of non-metric-invariant indicators, *p*_ni_

Across the simulation conditions, *p*_ni_ will either be 0, 2, or 4. For all indicators in Group 1, the factor loadings were set to 0.7, while some of those in Group 2 were set to 0.3 to represent moderate degree of metric non-invariance. This was similar to the conditions in some previous studies (Kaplan and George, 1995; Donahue, 2006).

#### Regression coefficients, γ

There were four levels of **γ**, two of which with equal regression coefficients ({0.1, 0.1} and {0.5, 0.5}) and two with them different ({0.5, 0.33} and {0.33, 0.5}). In the equal **γ** conditions the grouping variable did not moderate the effects of *F*_X_ on *F*_Y_, and Type I error rates were investigated. We were also interested in whether the effect of *F*_X_ being large (i.e., 0.5) and small (i.e., 0.1) influences Type I error rates. In the conditions with different **γ** the effects of *F*_X_ on *F*_Y_ were different for Group 1 and for Group 2, so there were moderation effects between groups and *F*_X_ on *F*_Y_ and powers of detecting the true moderation effects were investigated. The numbers were chosen based on the benchmark of small (γ = 0.1), medium (γ = 0.33), and large effects (γ = 0.5; Cohen, 1988).

#### Location of non-invariance

The metric non-invariance occurred either on only *F*_X_ or only *F*_Y_. Note that this design factor were not applicable to conditions with *p*_ni_ = 0.

#### Sample size, *N*

There were two levels of sample size: 200 and 500, in consistent with some previous studies (e.g., Yoon and Millsap, 2007).

Mplus 7.0 (Muthén and Muthén, 2012) was used to generate 500 data sets for each condition. All variables were assumed multivariate normally distributed. The two factor variances in Group 1 were 1.0 and those in Group 2 were 1.3. For Group 1, the unique factor variances of all indicators were set to 0.51 in the population, so that the invariant indicators had a variance of 1.0. The unique factor variances for Group 2 were set to 0.51 × 1.3 = 0.663 so that the proportion of explained variances for the invariant indicators was constant across groups. Because scalar invariance was not the focus of the present study and might not be required for correctly modeling moderation effects, all intercepts and factor means in the population were set to zero.

The data sets generated were then analyzed in Mplus. The analytic model was identified by fixing the factor loadings of the first indicators for *F*_X_ and for *F*_Y_ to the population value (i.e., 0.7), while allowing the latent factor variances of *F*_X_ and of *F*_Y_ to be freely estimated. Hence, both *F*_X_ and *F*_Y_ were scaled to the same unit as the population model and across replications, so that the **γ** values from the two groups were comparable. To identify the mean structure, the latent factor mean of *F*_X_ and the latent intercept of *F*_Y_ were fixed to zero for both groups, while the intercepts and the unique factor variances were allowed to be freely estimated without cross-group equality constraints, as scalar and strict invariance conditions were not assumed.

For conditions with *p*_ni_ = 0, the data sets were analyzed by fitting only the model with metric invariance. For other conditions with *p*_ni_ > 0, both the (misspecified) model with metric invariance and the (correct) model with partial metric invariance were fitted. Then for each data set, we obtained the point estimate of $\Delta \hat{\gamma }=\gamma_{1}-\gamma_{2}$ (using the MODEL CONSTRAINT command in Mplus) and the Wald test statistic (using the MODEL TEST command in Mplus) for the null hypothesis γ_1_ = γ_2_. Note that we also obtained the results for the likelihood ratio test, which is usually more accurate for finite samples, but we only presented the results for the Wald test as the two tests were nevertheless asymptotically equivalent and produced similar empirical powers and Type I error rates across simulation conditions.

The dependent variables of investigation for the simulations were the percentage of replications where the test statistics were statistically significant at 0.05 level and the standardized bias of $\Delta \hat{\gamma }$. If in the population, γ_1_ = γ_2_, then the percentage of replications with statistically significant Wald test statistic was the empirical Type I error rate (α^*^). Taking into account the sampling variability in 500 replications, an α^*^ between 3.4% and 7.3% is within the 95% confidence interval when the true Type I error rate is 5%. Empirical Type I error rates over the range of [3.4%, 7.3%] are defined as biased. We expected to see biased Type I error rates and the standardized biases to be large when metric invariance is incorrectly assumed.

If in the population γ_1_ ≠ γ_2_, the percentage where the test statistics were statistically significant at 0.05 level was the empirical power. Given that power is a function of effect size and sample size, the empirical power rates yielded from fitting the model with metric invariance in *p*_ni_ = 0 condition were treated as the baseline; those yielded with *p*_ni_ > 0 from incorrectly assuming measurement invariance and correctly assuming partial invariance models were then compared to the baseline. We expected to see power estimates from models incorrectly assuming measurement invariance were more different from the baseline then the correctly assuming partial invariance models.

Denote ${\hat{\gamma }}_{1}^{\left(i\right)}$ and ${\hat{\gamma }}_{2}^{\left(i\right)}$ as the estimated values of γ_1_ and γ_2_ for the *i*th replication, and ${\bar{\gamma }}_{1}$ and ${\bar{\gamma }}_{2}$ as the corresponding means across replications. The standardized bias (Collins et al., 2001) was computed as

$$\text{standardized bias}=\frac{\left({\bar{\gamma }}_{1}-{\bar{\gamma }}_{2}\right)-\left(\gamma_{1}-\gamma_{2}\right)}{\text{SD}\left({\hat{\gamma }}_{1}-{\hat{\gamma }}_{2}\right)},$$

where

$$\text{SD}\left({\hat{\gamma }}_{1}-{\hat{\gamma }}_{2}\right)=\sqrt{\frac{\sum_{i=1}^{R}{\left[\left({\hat{\gamma }}_{1}^{\left(i\right)}-{\hat{\gamma }}_{2}^{\left(i\right)}\right)-\left({\bar{\gamma }}_{1}-{\bar{\gamma }}_{2}\right)\right]}^{2}}{R}},$$

and *i* = 1, 2, …, *R* was the index of replications where *R* = 500. The standardized bias was the ratio of the average raw bias over the standard error of the sample estimator of the parameter, and a standardized bias with absolute value < 0.40 was regarded as acceptable (Collins et al., 2001).

### Result

The simulation results for the condition with null moderation effects were displayed in Table 1. When the measurement invariance assumption held on both the predictor and the outcome in population model (i.e., *p*_ni_ = 0), using the analytic model assuming measurement invariance across groups yielded unbiased moderation effect estimates and unbiased α^*^.

**Table 1:** Empirical type I error rate (in percentage) and standardized bias for study 1.

			**Non-invariance on** ***F***_**X**_				**Non-invariance on** ***F***_**Y**_			
			**Type I error (%)**		**Std. Bias (**$\Delta \hat{\gamma }$**)**		**Type I error (%)**		**Std. Bias (**$\Delta \hat{\gamma }$**)**	
***N***	**γ**	***p*_ni_**	**MI**	**pMI**	**MI**	**pMI**	**MI**	**pMI**	**MI**	**pMI**
200	{0.1, 0.1}	0	4.2	–	0.00	–	–	–	–	–
		2	4.2	4.6	−0.12	0.00	4.2	4.0	0.11	0.01
		4	6.0	4.6	−0.34	−0.01	5.4	4.2	0.33	0.02
	{0.5, 0.5}	0	4.6	–	0.01	–	–	–	–	–
		2	8.0	4.4	−0.64	0.00	7.8	4.2	0.62	0.02
		4	35.2	4.0	−1.60	−0.01	38.8	4.8	1.74	0.02
500	{0.1, 0.1}	0	4.6	–	0.01	–	–	–	–	–
		2	5.4	5.2	−0.18	0.01	5.0	5.2	0.19	0.01
		4	7.6	5.0	−0.51	0.01	7.0	5.0	0.52	0.00
	{0.5, 0.5}	0	4.0	–	−0.01	–	–	—	–	–
		2	14.4	3.6	−1.06	−0.01	13.8	4.8	0.97	−0.01
		4	77.8	3.2	−2.79	−0.01	77.8	5.2	2.74	−0.01

*p_ni_, number of non-metric-invariant indicators; **γ**, population regression coefficient of F_Y_ on F_X_; MI, analytic model assumed metric invariance; pMI, analytic model correctly assumed partial metric invariance; Std. Bias, standardized bias = $\Delta \hat{\gamma }/\text{SD}\left(\Delta \hat{\gamma }\right)$, where SD(Δ$\hat{\gamma }$) is the standard deviation of the differences in the estimated γs across all replications*.

When the non-invariance occurred on *F*_X_, as partial metric invariance was the correctly specified model, with a partial invariance model α^*^ was close to the 0.05 nominal significance level and the moderation effect was estimated with absolute values of standardized bias <0.02 (< 0.40 as acceptable). On the other hand, α^*^ was inflated when metric invariance were falsely assumed. The difference between α^*^ from the nominal level increased as one or more of *p*_ni_, *N*, and the values of **γ** increased. For example, when *N* = 200, *p*_ni_ = 2, and **γ** = {0.1, 0.1}, α^*^ = 4.2%; when *N* = 500, *p*_ni_ = 4, and **γ** = {0.1, 0.1}, α^*^ = 7.6%; and when *N* = 500, *p*_ni_ = 4, and **γ** = {0.5, 0.5}, α^*^ = 77.8%. An analysis of variance (ANOVA) including *N*, **γ**, and *p*_ni_ showed that *p*_ni_ produced the largest impact on α^*^ (η^2^ = 0.34), followed by **γ** (η^2^ = 0.21) and *N* (η^2^ = 0.04). The bias of the estimated values of Δγ followed a similar pattern. For instance, With *N* = 500, *p*_ni_ = 4, and **γ** = {0.5, 0.5}, the standardized bias of the null moderation effects was −2.79, which was a substantial bias.

The pattern of α^*^ and the absolute values of the standardized bias when non-invariance occurred in *F*_Y_ was very similar to those when non-invariance occurred in *F*_X_. However, the sign of the standardized bias was reversed, which means that when non-invariance occurred in the outcome's structure, the moderation effects were overestimated. Considering both the locations of the non-invariance, we found that using models that incorrectly assumed measurement invariance would result in substantially biased moderation effect estimate and inflated Type I error rate.

Table 2 showed the results of both the powers and standardized biases with nonzero moderation effects. When the non-invariance occurred on *F*_X_, the corrected partial metric invariance models performed well as they showed no bias on the moderation effect estimates with standardized biases from −0.03 to 0.01. On the contrary, the metric invariance model yielded biased estimates of the moderation effects and the influence was more salient as both *N* and *p*_ni_ increased. For example, when **γ** = {0.5, 0.33}, the standardized bias was −0.43 with *N* = 200 and *p*_ni_ = 2; the standardized bias increased to −1.84 with *N* = 500 and *p*_ni_ = 4. An ANOVA showed that *p*_ni_ produced the largest impact on the biased moderation estimates (η^2^ = 0.79), followed by *N* (η^2^ = 0.09) and **γ** (η^2^ = 0.01).

**Table 2:** Empirical power (in percentage) and standardized bias for study 1.

			**Non-invariance on** ***F***_**X**_				**Non-invariance on** ***F***_**Y**_			
			**Power (%)**		**Std. Bias (**$\Delta \hat{\gamma }$**)**		**Power (%)**		**Std. Bias (**$\Delta \hat{\gamma }$**)**	
***N***	**γ**	***p*_ni_**	**MI**	**pMI**	**MI**	**pMI**	**MI**	**pMI**	**MI**	**pMI**
200	{0.5, 0.33}	0	32.2	–	−0.01	–	–	–	–	–
		2	16.0	30.8	−0.43	−0.02	53.0	31.2	0.48	0.00
		4	4.4	31.2	−1.12	−0.03	81.8	29.4	1.29	0.02
	{0.33, 0.5}	0	33.0	–	0.02	–	33.0	–	–	–
		2	50.0	30.8	−0.61	0.01	15.8	30.0	0.52	0.03
		4	77.0	26.8	−1.57	−0.01	4.2	27.4	1.52	0.04
500	{0.5, 0.33}	0	70.2	–	−0.01	–	–	–	–	–
		2	33.8	67.2	−0.69	−0.02	89.0	67.4	0.76	−0.01
		4	5.0	62.4	−1.84	−0.02	99.2	62.4	2.01	−0.02
	{0.33, 0.5}	0	67.6	–	0.00	–	–	–	–	–
		2	90.6	67.2	−1.01	−0.01	36.0	66.2	0.79	0.00
		4	99.4	59.4	−2.71	−0.01	5.0	62.8	2.38	0.00

*p_ni_, number of non-metric-invariant indicators; **γ**, population regression coefficient of F_Y_ on F_X_; MI, analytic model assumed metric invariance; pMI, analytic model correctly assumed partial metric invariance; Std. Bias, standardized bias = $\Delta \hat{\gamma }/\text{SD}\left(\Delta \hat{\gamma }\right)$, where SD(Δ$\hat{\gamma }$) is the standard deviation of the differences in the estimated γs across all replications*.

In terms of the powers for detecting the moderation effects, the corrected partial invariance model yielded powers around 30% and 60% for *N* equals 200 and 500, respectively. Such power estimates were close to population model with the measurement invariance assumption held (33% for *N* = 200 and 70% for *N* = 500). On the other hand, if metric invariance was falsely assumed, there was a substantial decrease in powers for the conditions where non-invariance occurred. For example with **γ** = {0.5, 0.33}, *N* = 500, *p*_ni_ = 2, and non-invariance on *F*_X_, the empirical power was half as would be obtained when metric invariance held in the population (33.8% vs. 70.2%); with **γ** = {0.33, 0.5}, *N* = 200, *p*_ni_ = 4, and non-invariance on *F*_Y_, the empirical power was only 1/8 as the power would be obtained when metric invariance held in the population (4.2% vs. 33.0%).

Note that power loss was detected as both the *N* and *p*_ni_ increased when the non-invariance occurred on *F*_X_ and **γ** = {0.5, 0.33}; simulation conditions related to non-invariance occur-ed on *F*_Y_ and **γ** = {0.33, 0.5} would lead to inflated power estimates as both the *N* and *p*_ni_ increased. The main reason for different patterns on the power estimates were that when the factor loadings of Group 1 (0.7) was larger than those of Group 2 (0.3) in the presence of non-invariance on *F*_X_, the estimated moderation effect was negatively biased, whereas when non-invariance occurred in *F*_Y_, the estimated moderation effect was positively biased. Additionally, the true moderation effect was −0.17 when **γ** = {0.33, 0.5}; therefore, the negative biases caused by falsely assuming measurement invariance would result in more negative moderation effects estimates and inflated power.

## Study 2

**Figure 2:**
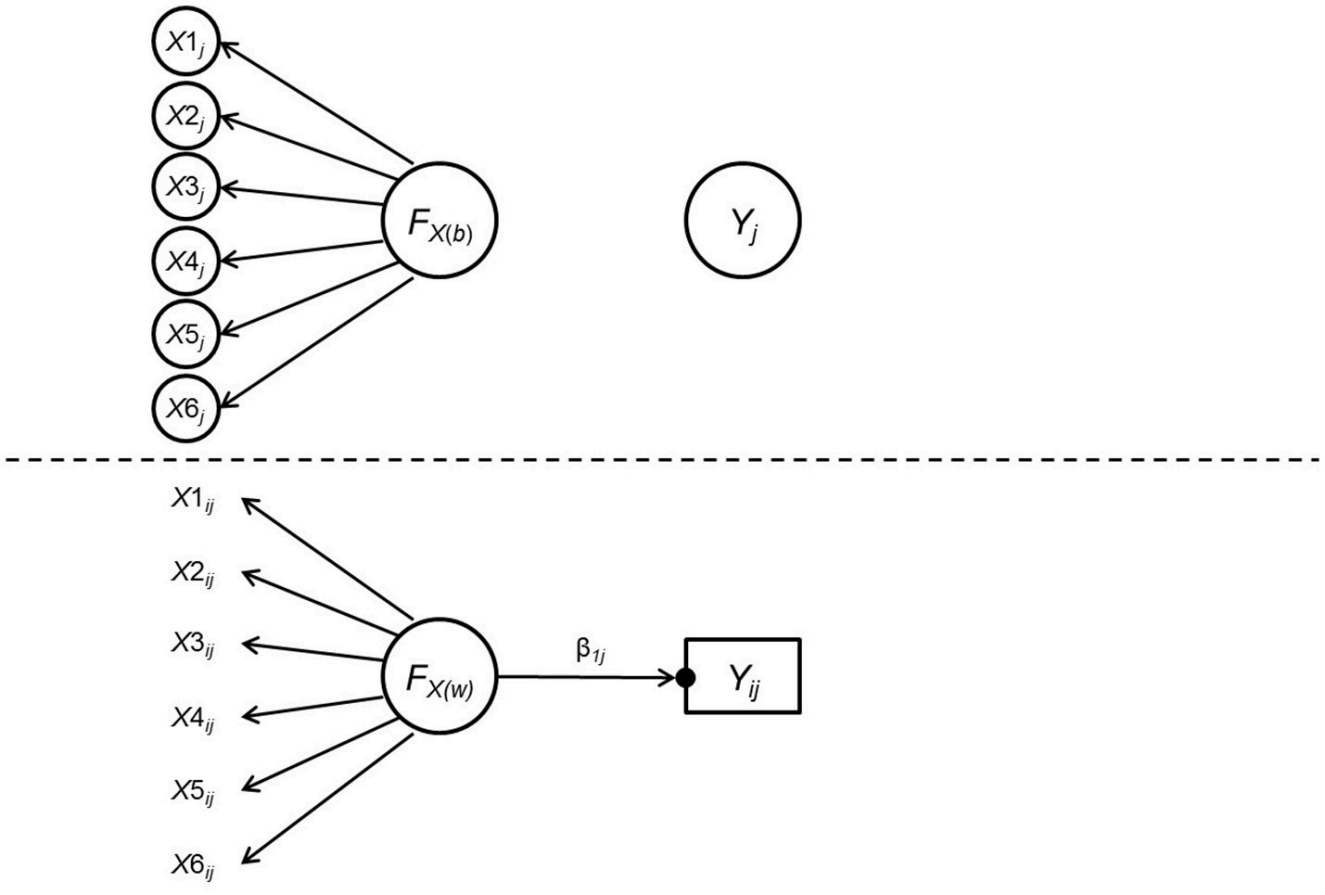
Data generating model for Study 2. *F*_*X*(*w*)_ and *F*_*X*(*b*)_ are the latent predictor variable at the within-level and the between-level, respectively. *Y*_*ij*_ and *Y*_*j*_ are the within-level and the between-level components of the outcome variable *Y*. β_1*j*_ = within-level regression coefficient of *Y* on *F*_*X*(*w*)_, whose magnitude varies across clusters as indicated by the black dot. Conditioning on the grouping variable, measurement invariance was assumed across clusters such that the within-level and the between-level factors loadings were identical, and that there were no residual variances for the six indicators at the between-level.

In Study 2, we aim to extend the scope of the MI-moderation relation to multilevel data. We focused on how the measurement (non-)invariance across groups at the between level influences the test of cross-level moderation effect, which was one of the prevailing issues among social and behavioral research. Specifically, we used the data generating model shown in Figure 2, which was one of the simplest models including multilevel measurement (non-)invariance and a within-level predictor, to depict the cross-level moderation effect. As can be seen in Figure 2, the latent predictor was measured by six indicators and the cross-level interaction effect was denoted by the difference between the within-level path coefficient from the predictor to the outcome across groups. It was assumed that the predictor did not have an effect on the outcome in the between level.

Because multilevel data are usually of larger sample size, we expect the impact of multilevel non-invariance on the Type I error rate and power to be bigger. In addition, we are interested in whether the impact varies across multilevel specific design factors such as the intraclass correlation (ICC), number of clusters, and cluster size. Because in Study 1, we found that different locations of non-invariance mainly resulted in changes in signs of the biases of the moderation effects, in Study 2 we only focused on measurement non-invariance on the predictor side. Likewise, we only consider the positive moderation effects condition in Study 2 given that negative moderation effect led to similar results in biases in Study 1. A second Monte Carlo simulation study was conducted, as described below.

### Monte carlo simulation

The study had a 2 (*p*_ni_) × 2 (**γ**) × 2 (ICC, intraclass correlation) × 2 (*m*, number of clusters) × 2 (*c*, cluster size) design. In each condition there were two groups (Group 1 and Group 2), and sample sizes (both within and between) were assumed equal across groups. The latent predictor, *F*_X_, had the same six-indicator measurement structure in both the within and the between level as in Study 1; the observed outcome, *Y*_*ij*_, of the *i*th observation in the *j*th cluster, contained no measurement error and was assumed measurement invariant. The model can be expressed as

$$\begin{array}{l} \text{Within level:}Y_{ijg}=Y_{\left(b\right)jg}+\gamma_{10g}F_{\text{X}\left(w\right)ijg}+r_{ijg},\\ {\text{X}}_{ijg}={\text{X}}_{\left(b\right)jg}+\lambda_{\left(w\right)g}F_{\text{X}\left(w\right)ijg}+\delta_{\left(w\right)ijg};\\ \text{Between level:}Y_{\left(b\right)jg}=\gamma_{00g}+\zeta_{jg},\\ {\text{X}}_{\left(b\right)jg}=\alpha_{g}+\lambda_{\left(b\right)g}F_{\text{X}\left(b\right)jg}+\delta_{\left(b\right)jg}, \end{array}$$

where $\text{X}={\left[X_{1},X_{2},\ldots \right]}^{\prime }$ was a vector containing the observed values of the indicators, and their group means comprised **X**_(*b*)_. The vectors **λ**_(*w*)_ and **λ**_(*b*)_ contained the within-level and between-level factor loadings, respectively. In this study we assumed that **λ**_(*w*)*g*_ = **λ**_(*b*)*g*_ = **λ**_*g*_. In the within level, *F*_X(*w*)_ (the within-level exogenous factor) had an effect of magnitude γ_10*g*_ on *Y*, where *g* is the group index. In the between level, *F*_X(*b*)_ (the between level exogenous factor) had no effect on *Y*. Note that there were no between-level random effects on γ_10*g*_ and on the factor loadings. We also assumed measurement invariance across clusters, implying that **δ**_(*b*)*jg*_ = **0** and homogeneous **δ**_(*w*)*jg*_ across clusters (Jak et al., 2013), in addition to **λ**_(*w*)*g*_ = **λ**_(*b*)*g*_. The design factors were described below.

#### Number of non-metric-invariant indicators, *p*_ni_

*p*_ni_ was either 0 or 2 out of the six indicators of *F*_X_. Whereas two of the factor loadings were always set to 0.7 in Group 1, for conditions with *p*_ni_ = 2, those loadings were set to 0.3 in Group 2. The factor loadings for other four indicators were 0.7, 0.3, 0.5, and 0.6, for both the within level and the between level.

#### Regression coefficients, γ

There were two levels of **γ**: {0.3, 0.2} (moderation present) and {0.3, 0.3} (moderation absent).

#### Intraclass correlation, ICC

Based on previous simulations (Kim et al., 2012), in this study there were two levels of ICC: 0.10 and 0.35, representing small and large within-cluster correlations for the latent variable *F*_X_ and for the outcome *Y*.

#### Cluster size, *c*

Based on previous literature (Clarke, 2008; Kim et al., 2012), there were two levels of cluster size: 5 and 20, representing small and medium number of observations within a cluster. For simplicity we generated data with all clusters having the same size in both groups.

#### Number of clusters, *m*

Hox and Maas (2001) suggested the number of groups larger than 100 as the minimum requirement for yielding accurate multilevel regression estimates. Later on, Maas and Hox (2005) found groups number equal to 30 could also yield accurate multilevel regression estimates. McNeish (2017) did a literature review on 70 multilevel studies and found 90% of them fail to meet Hox and Maas's criterion of 100 clusters, and that the median number of clusters was 44. In the present study, we specified the number of clusters in each group either 30 or 100, representing the small and large number of clusters.

Mplus 7.0 was used to generate and analyze (with ESTIMATOR=MLR) 500 data sets for each condition. All exogenous variables and random effects were assumed multivariate-normally distributed. For both groups the variances of *F*_X(*w*)_ and *Y* both equaled to 1.0, and that of *F*_X(*b*)_ and ζ_*jg*_ were functions of the ICC. The variance of **δ**_(*w*)*ijg*_ was set to 0.5**I** so that the level-1 unique factor variances were similar in values to those in Study 1 (i.e., 0.51 in Study 1 when the latent factor variance is one). The covariance and mean structure were identified similarly as in Study 1 by fixing the factor loadings of the first indicators for *F*_X(*b*)_ to the population value and the latent mean of *F*_X(*b*)_ to zero for both groups. Additionally, within the same group the factor loadings were constrained to be equal in the between and the within levels so that metric invariance was assumed across clusters (Jak et al., 2013). Because scalar invariance was not the focus of the present study and may not be required for correctly modeling moderation effects, all intercepts and factor means in the population were set to zero.

The dependent variables of investigation were the standardized biases and the rejection rates of the Wald test statistics for the difference in γ_10_, which reflected either the empirical Type I error rate (α^*^) or the empirical power, and were obtained in the same manner as in Study 1. Also as in Study 1, for conditions with *p*_ni_ = 2, both the metric invariance model and the partial metric invariance model were fitted. We expected that model falsely assuming measurement invariance would lead to biased moderation estimation, inflated Type I error rate (when *p*_ni_ = 0), and power more different from the baseline (when *p*_ni_ = 2).

### Result

Results for Study 2 were shown in Table 3. In the conditions absent of moderation effects (**γ** = {0.3, 0.3}), fitting data with a metric invariance model when the true population model followed the measurement invariance assumption (*p*_ni_ = 0) led to unbiased moderation effect estimates and unbiased α^*^, regardless of the level of ICC, *m*, and *c*. The same pattern was observed while employing the corrected partial metric invariance model to fit data from a measurement non-invariance population, as such practice also led to unbiased moderation estimates and α^*^ close to the 5% nominal significance level across different ICC, *m*, and *c* simulation conditions.

**Table 3:** Empirical type I error rate, power, and standardized bias for study 2.

				**γ** = {0.3, 0.3}				****upgamma**** = {0.3, 0.2}			
				**Type I Error (%)**		**Std. Bias (**$\Delta \hat{\gamma }$**)**		**Power (%)**		**Std. Bias (**$\Delta \hat{\gamma }$**)**	
**ICC**	***p*_ni_**	***m***	***c***	**MI**	**pMI**	**MI**	**pMI**	**MI**	**pMI**	**MI**	**pMI**
0.10	0	30	5	6.60	–	0.01	–	15.00	–	0.01	–
			20	7.20	–	−0.02	–	34.80	–	−0.02	–
		100	5	5.80	–	0.02	–	31.20	–	0.02	–
			20	6.00	–	0.04	–	81.00	–	0.03	–
	2	30	5	9.00	5.80	−0.55	0.01	7.00	14.20	−0.41	0.01
			20	25.60	7.00	−1.17	−0.02	10.00	34.40	−0.91	0.02
		100	5	16.40	6.40	−1.07	0.01	4.80	28.60	−0.81	0.02
			20	55.20	5.80	−2.13	0.03	16.80	79.20	−1.60	0.03
0.35	0	30	5	6.20	–	0.02	–	15.20	–	0.02	–
			20	7.40	–	−0.02	–	35.20	–	−0.03	–
		100	5	5.40	–	0.01	–	30.80	–	0.02	–
			20	5.40	–	0.03	–	80.60	–	−0.03	–
	2	30	5	7.60	5.20	−0.53	0.02	6.80	14.60	−0.39	0.02
			20	25.20	7.20	−1.16	−0.02	10.20	32.80	−0.91	−0.02
		100	5	15.80	5.80	−1.04	0.01	4.60	25.80	−0.79	0.02
			20	54.60	5.40	−2.13	0.03	18.20	78.20	−1.61	0.03

***γ**, population regression coefficient of F_Y_ on F_X_; ICC, intraclass correlation; p_ni_, number of non-metric-invariant indicators; m, number of clusters; c, number of observations in a cluster; MI, analytic model assumed metric invariance; pMI, analytic model assumed partial metric invariance; Std. Bias, standardized bias = $\Delta \hat{\gamma }/\text{SD}\left(\Delta \hat{\gamma }\right)$, where SD(Δ$\hat{\gamma }$) is the standard deviation of the differences in the estimated γs across all replications*.

When non-invariance occurred (*p*_ni_ = 2), fitting data with a metric invariance model yielded substantially underestimated moderation effect and inflated α^*^. Such trend became more salient as *m* and *c* increased. For example with ICC = 0.10, *p*_ni_ = 2, *m* = 30, and *c* = 5, the standardized bias was −0.55 with α^*^ of 9%; with ICC = 0.10, *p*_ni_ = 2, *m* = 100, and *c* = 20, the standardized bias increased to −2.13 with α^*^ of 55.2%. An ANOVA analysis on the standardized bias with *p*_ni_, *m*, *c*, and ICC showed that *p*_ni_ had the largest impact on estimation biases (η^2^ = 0.70), followed by *c* (η^2^ = 0.08), *m* (η^2^ = 0.06), and ICC (η^2^ close to 0). ICC showed no impact of falsely assuming metric invariance on yielding biased moderation estimates and inflated α^*^. For example with ICC = 0.10, *p*_ni_ = 2, *m* = 100, and *c* = 5, the standardized bias was −1.07 with α^*^ of 16.4%; increasing the ICC to 0.35 while keeping the other design factors to be the same led to similar results with standardized bias = −1.04 and α^*^ = 15.8%.

In the conditions with nonzero moderation effect (**γ**: {0.3, 0.2}), again, employing the correctly specified partial invariance model resulted in unbiased moderation effect estimates. On the other hand, falsely assuming metric invariance led to substantially underestimated moderation effects across simulation conditions with standardized biases from −0.39 to −1.61. Consistent with the null moderation condition, an ANOVA analysis on the standardized bias indicated *p*_ni_ had the largest impact on estimation biases (η^2^ = 0.70), followed by *c* (η^2^ = 0.10), *m* (η^2^ = 0.06), and ICC (η^2^ close to 0). There was also a substantial loss in power when fitting a metric invariance model to data draw from a population with non-invariance. For example, the power of the simulation condition of ICC = 0.10, *p*_ni_ = 0, *m* = 100, and *c* = 20 was 80% but it dropped to 16.8% when ICC = 0.10, *p*_ni_ = 2, *m* = 100, and *c* = 20. Again, ICC only had a trivial effect on the deflation of power.

## Discussion

In the literature, the impact of measurement invariance on testing moderation effects has not been fully examined. The ratio of the non-invariant items have been found to be an important factor on the estimation accuracy of the path coefficients by the moderating groups (e.g., Guenole and Brown, 2014). In much of previous work, the focus was limited to single level data structure, without considerations of nested data structure. Additionally, the direct statistical test of the moderation effect was largely ignored in previous research. The current study investigated the impact of partial measurement invariance, with a focus on the metric invariance, on the estimation and testing of moderation effects on both single and multilevel structures, in terms of standardized bias, power and Type I error rate.

The results suggest that incorrectly assuming metric invariance holds while estimating moderation effects would lead to biased estimates. The impact is more salient as the number of non-invariant items increases, which is consistent with Guenole and Brown (2014)'s and Shi et al. (2017)'s findings with direct effects. On the other hand, fitting models correctly assuming partial metric invariance yielded accurate estimates regardless of samples size, main effects, number of non-invariant items, and the location of the non-invariance occurred.

In testing null moderation effects (i.e., γs are equal between two groups), the high Type I error rate yielded from falsely assuming metric invariance is not only related to the non-invariant item ratio but the magnitude of the main effects. These results suggest that evaluation of measurement invariance is of more importance when the main effect of the predictor is larger. On the other hand, the Type I error rates were on or below 5% with models correctly assuming partial metric invariance. Thus, before examining moderation effects, the metric invariance assumption should not be presumed without conducting any invariance test, even for cases in which the moderation tests turn out to be non-significant.

The location of the non-invariance (predictor vs. outcome) is associated with the direction of the biases of the moderation effects. In our simulations, all of the non-invariance conditions were specified such that factor loadings of Group 1 were equal or larger than those of Group 2. As evident from the simulation results, under such settings ignoring predictor non-invariance leads to underestimation of the moderation effects, whereas ignoring outcome non-invariance results in overestimated moderation effects. Our findings are consistent to Chen (2008) and Guenole and Brown (2014), in which they found that non-invariance on the predictor with lower factor loadings in group 2 would lead to underestimated path coefficient in group 1 (γ_1_) and overestimated path coefficient in group 2 (γ_2_). Hence, the moderation effect (γ_1_ − γ_2_) would likely be underestimated. On the other hand, non-invariance on the outcome changes the association to the opposite direction and results in the overestimation of the moderation effects.

Compared with models correctly assuming partial metric invariance, models falsely assuming metric invariance yielded moderation test with statistical power varying substantially. Taking into account the signs of the moderation effects, when the moderation effects are positive, ignoring non-invariance on the predictors leads to power loss, but ignoring non-invariance on the outcomes leads to increased power (at the cost of highly inflated Type I error rate). Likewise, an opposite association between the location of non-invariance and power is observed when the moderation effects are negative. Therefore, the increase in power in half of our simulation conditions in Table 2 is actually a byproduct of sacrificing the estimation accuracy of the moderation effects (i.e., overestimation). Ignoring non-invariance and resulting in power gain or loss depends on (a) the location of the non-invariance, (b) the signs of the moderation effects. Overall, it is not recommended to fit a model assuming metric invariance when the assumption is actually violated, even though it may increase the power of the moderation test.

There is also prospective evidence that falsely assuming multilevel metric invariance across groups has a negative impact on the estimation of the cross-level moderation effects, which leads to either substantially inflated Type I error rate or inflated/deflated statistical power of the moderation test. Both increases in *m* and in *c*, or in other words an increase in the total sample size, resulted in bigger problems in the estimation accuracy as well as α^*^ and power. Thus, for multilevel data, even with only one-third of the indicators being non-metric-invariant, tests of moderation can become hugely misleading.

Across simulation conditions, the number of non-invariant items played a huge role in influencing the performance of the moderation estimates. Researchers use multilevel data with a number of clusters (e.g., number of classrooms) larger than 100 or cluster size larger than 20 (e.g., 20 students in each classroom) should be particularly cautious about the negative impact of non-invariant items. Additionally, the cluster size (*c*) seemed to have a larger impact than the number of clusters (*m*) when falsely assuming measurement invariance in the moderation estimation. Intraclass correlation (ICC) was trivially related to the moderation estimates, probably because the path of interest was defined in the within-level. On the contrary, previous research has shown that ICC is highly related to between-level analysis (Kim et al., 2012). Thus, one potential explanation for the discrepancy is that, given that the cross-level moderation coefficients were mainly defined in the within-level, the level of data dependency has less influence on the moderation effect estimates.

Findings from Study 1 and Study 2 highlight the importance of testing metric invariance before conducting a moderation test with both single and multilevel data structure. If the metric invariance assumption is violated, a partial metric invariance model in which the non-invariant factor loadings between groups are correctly reflected should be employed. Researchers should also be aware that the MI-moderation relationship is highly affected by the ratio of non-invariant items in the scale and the overall sample size. Overall, while testing moderation effects in a multiple-group analysis setting, we recommend the test of measurement invariance for both the predictors and outcomes by the moderator groups. If the measurement invariance assumption holds, then employing models with such an assumption implied is appropriate. On the other hand, if the measurement invariance assumption is violated, then the use of a corrected partial invariance model would yield more accurate estimates and unbiased Type I error rates or power.

Some limitations and future study directions should be addressed. First, the research scenario only focused on metric invariance (i.e., invariance of the factor loadings). In practice, non-invariance may exist in the intercepts, factor loadings, unique factor variances, or some combinations of them. Previous simulation studies on latent growth modeling have shown that ignoring intercept non-invariance only leads to biased factor mean (or intercept) estimates (Kim and Willson, 2014). Research on multiple-group analysis also showed that ignoring intercept non-invariance has less impact on the prediction bias of the path coefficient in each group (Guenole and Brown, 2014). Therefore, we suspect that the impact of intercept non-invariance on the moderation effect estimates should be much smaller than that of factor loading non-invariance, but more conclusive evidence needs to be obtained from future methodological inquiries.

Second, for Study 2 we only tested cross-level moderation in the present study, but moderation effects may also occur at the between level, in which factors such as ICC may play a more important role in affecting the moderation estimates. Lastly, in the simulation, the indicators were assumed to be continuous and normal distributed when conditioned on the latent factors. It is important to see how measurement non-invariance with skewed and categorical indicators influence the estimation of the moderation effects. Therefore, future study can investigate the impact of falsely assuming measurement invariance under more complicated research settings.

## Author contributions

Y-YH led the implementation and manuscript writing of the study. ML designed and conducted the simulation. Both authors (Y-YH and ML) contributed to the design, analysis, interpretation of data, writing, and revising of the manuscript.

### Conflict of interest statement

The authors declare that the research was conducted in the absence of any commercial or financial relationships that could be construed as a potential conflict of interest.

## Footnotes

^1^One prerequisite to interpret moderation effects in the presence of non-invariance is that the constructs being measured are still conceptually comparable across groups (i.e., configural invariance). If the predictor and/or outcome represent different measurement structure across groups, the computed moderation effect may not be meaningful.

^2^Throughout this article, we investigated the multilevel measurement (non)invariance across an explicitly defined grouping variable, not the non-invariance for the between-versus within-level. Additionally, we assumed that the outcome variable is defined at the within-level but not at the between-level. Finally, the grouping variable was defined at the between-level but not the within-level in the multilevel case. All the mentioned conditions not investigated in the present study may have different implications that are also worthy of future studies.
